# Department of Bacteriology and Dermatology

**Published:** 1892-10

**Authors:** Frank J. Thornbury

**Affiliations:** Demonstrator of Bacteriology, Medical and Dental Departments, University of Buffalo


### DEPARTMENT OF BACTERIOLOGY AND DERMATOLOGY

Conducted by FRANK J. THORNBURY, M. D.,

Demonstrator of Bacteriology, Medical and Dental Departments, University of Buffalo.

### TETANUS BACILLUS.

THE tetanus bacillus seems to have contributed its share of ptomaines to the group of germ products. Breger has now isolated four toxines of tetanus. They are designated, tetanin, tetanotoxin, spasmatoxin, and hydrochlorotoxin. All of these are capable, in infinitely small doses, of producing typical tetanic spasms.

Work in the production of immunity is becoming very interesting. We have recently reported from the Innsbruck surgical clinic, another case of tetanus, cured by the hypodermic injection of 0.15 G to 0.20 G doses, Tizzoni & Catlani's antitoxin. This antitoxin was obtained from the blood serum of a dog, rendered artificially immune to tetanus.

Explanation of many previously obscure points is found in the results of bacteriological investigation. We are now in position to understand why it is that tetanus only occurs, as a rule, in deep wounds,—lacerated or punctured,—and particularly in toy pistol injuries, and punctures produced by splinters from the floor.

The tetanus bacillus, being anerobic, will not develop in presence of air, and, therefore, only produces its morbid consequences when buried deep in the tissues. Only by observance of these biological peculiarities can we obtain pure cultures of the bacillus. The exclusion of air from the culture medium is accomplished by passing hydrogen gas through it, or the agar tube is inoculated by introducing the needle deep into the interior of the agar.

Of course, there is no difficulty in understanding how the bacilli gain entrance to the wound. Garden earth abounds in tetanus bacilli, and pure cultures may be obtained from this source by inoculating mice with the earth, and as they develop tetanus, pouring plates from the blood, and thus isolating the bacillus.

On a certain island near New York, nearly every wound of any depth is complicated by tetanus, and the soil here is found to be especially rich in tetanus bacilli.

---

### SPORE AND NON-SPORE FORMING PATHOGENIC BACTERIA.

By reason of the widely varying degrees of resistance between the ordinary vegetative bacteria and those forming spores, it is very important that we should be familiar with the pathogenic micro-organisms which have this special power of resistance.

The list is briefly as follows: 1.—Spore forming pathogenic organisms, (*a*) anthrax bacillus, (*b*) tetanus bacillus, (*c*) tubercle bacillus, (*d*) influenza bacillus, probably. 2.—Non-spore forming pathogenic organisms, (*a*) staphylococcus pyogenes—aurius albus and citrus, (*b*) streptococcus pyogenes, (*c*) streptococcus of erysipelas, (*d*) Löffler's diphtheria bacillus, (*e*) Löffler-Schutz glanders bacillus.

---

### LICHEN RUBOR PLANUS, COMPLICATED BY PEMPHIGUS EXFOLACIUS.

This extraordinary case which occurred during the past winter in the dermatological clinic of Vienna, is said by Prof. Kaposi to be the only case of the kind on record; at least no analogous instance is found in the literature. The case was one of ordinary lichen rubor planus in a man about sixty. He had been in the hospital twice previous for treatment of his lichen rubor, which had now recurred and presented the usual characteristic, small glistening areas, slightly smaller than a hypodermic tablet, concentrically depressed and distributed quite symmetrically over the extensor surfaces of the extremities and over the anterior and lateral thoracic regions. The usual arsenical treatment was being carried out when the patient suddenly became extremely ill, and was attacked with a pemphigus universalis.

Locally, there was tenderness and hyperemia with some vesiculation and later large bleb formation. The latter were present, especially over the body and upper extremities, and in parts where the atrophic lichen papules were undergoing involution. The blebs contained an opalescent serum ; after their spontaneous or artificial rupture and escape of the fluid, exfoliation of the superficial epidermal lagers occurred and left exposed a raw, reddened, and extremely painful surface.

Constitutionally, the man was affected as though suffering from some acute infectious disease. As stated above, he was completely and quite suddenly prostrated, and had chills followed by elevation of temperature to 102° or 103°. The pulse was 112 to 120. There was complete anorexia and insomnia on account of pain.

The countenance wore an expression of anxiety and distress, every movement of the body caused intense suffering, and, generally speaking, the patient was in a very miserable condition. Kaposi presented the case before the Vienna Dermatological Society, and it was discussed by Hebra, Ehrmann, Neumann, Lang, Finger, and others, but none of these so widely experienced dermatologists had ever seen an analogous case.

The treatment instituted was symptomatic, and comprised, locally, the Kaposi water baths and later the tar baths; constitutionally supportive remedies were administered in the form of tonics and free stimulation. The prognosis seemed unfavorable. Two other cases in the ward died of pemphigus vulgaris about the same time; but the case here referred to, began to improve after a long and critical illness, and he was canvalescent in Ward 33, of Kaposi's clinic, at the last knowledge of the writer.

---

### THE HYPODERMATIC TREATMENT OF SYPHILIS.

This method is quite generally in vogue on the Continent at the present time, and it has certain merits over the use of enunctions and the giving of mercury internally. First of all is the infinitely less trouble associated,—a thing always of importance to the patient. The difference consists in a hypodermic injection once in eight days, as compared with an enunction for quarter or half of an hour every day, or the taking of medicine every three or four hours for months.

The results are very satisfactory, especially in the secondary stage. Here the eruption subsides quite symmetrically under influence of the treatment. The gluteal region is the part usually selected for the injection, which is made as deeply as the length of the needle will permit. A variety of solutions are employed by the Vienna sypholographers, each one having his special formula. Kaposi prefers an aqueous solution of corrosive sublimate in chloride of sodium. Finger uses the salicylate of mercury, while Neumann and Ehrmann inject the concentrated oleum senerium. Prof. DeAmices lauds the superior merits of succinimide of mercury in a one per cent. solution of cocaine. Very satisfactory results are produced by the latter in the tertiary as well as the secondary stages. Economy of time, then, and convenience, with evident prompter action and more immediate results, are the arguments for this method of treatment, and those which have gained for it the popularity which it now enjoys.

In clinic practice a number of cases can be dispensed with in a comparatively short time. On the day for syphilitics in Lassar's clinic, Berlin, thirty to fifty victims present themselves in line before the assistant, who injects them as fast as they can conveniently change places in front of him. After injection the patient applies gentle massage for two or three minutes with a small quantity of sterilized vaseline over the gluteal area. The hypodermics are administered alternately in the right and left sides. It is seldom that any induration occurs. I have never seen abscess resulting.

---

### THE BACILLUS OF RHINOSCLEROMA.

Rhinoscleroma, first described by Hebra, is an affection characterized by a chronic inflammatory thickening and induration of the tissues, by a slow and insidious process of infiltration. Usually, the nasal appendage, fauces, and larynx are the seat of the trouble, although other parts may be involved.

The etiology of the disease has always been a question of much interest. Kaposi, in a report of nine cases, first showed that the disease had an infectious origin in a special bacillus; and now Paltauf (*Hygienische Rundschau,*) comes forward with fifteen additional cases, in which he corroborates Kaposi's observations, in the announcement that the bacillus is invariably present and permits of very precise differentiation from the former Friedlander pneumococcus, which it was thought very closely to similate. In each of the fifteen cases reported, Paltauf succeeded in cultivating the rhinoscleroma bacillus from the blood and tissues in which, hystologically examined, vast numbers of the bacilli were present. According to the literature, there have been in all now about forty-two cases of rhinoscleroma, in which the bacilli has been demonstrated by cultivation.

The constant presence of the organisms can leave no further doubt as to their causative relation to the disease. Inoculation of the bacillus by Stapanow, in the eyes of guinea-pigs, produced a diffuse keratitis and tubidity of the aqueus. The bacteria were found in the iris, cornea, and lens, and produced a hyeline degeneration of these structures. A bacteriological may now be added to the therapeutic test in differentiating this disease from lues. In addition to the cases affecting the nose and alae nasi, Paltauf found that the gums, fauces, trachea, and larynx are frequently the seat of rhinoscleroma, and that the disease is ofen primary in, and confined to, the larynx and trachea.

---

### CHOLERA.

ACCORDING to M. Netter (*Le Progrès Médical*), the bacillus of the present epidemic differs slightly from the comma bacillus discovered by Koch, in India, in 1883. It is thicker, shorter, and larger than the Indian bacillus, causes turbidity of bullion, and in pancreas prepared gelatin it grows more rapidly. The bacillus was found in the dejections in twenty-nine cases of cholera. In one case of bronco-pneumonia it was also found in the sputum, but it is never present in the blood. In thirty additional cases examined in Paris, the comma bacillus was *not* found. These cases were probably cholera nostras, as there has been, as yet, no authentic case of Asiatic cholera in which the comma bacillus was not present (Fraenkel).

The question of a spore in the cholera bacillus is an important one for obvious reasons, but, as far as the majority of observers have been able to determine, the comma bacillus has no spore, although Huppe, of Prague, claims a fructification propensity of this germ by virtue of an arthrosporulation, which process he claims to have observed under the microscope. First, the bacilli grow to form spiral threads, then occurs in places refractive nuclei, each of which give rise to two or more immotile embryonic cells ; the latter do not multiply by the usual method of spontaneous division, but by a process of proliferation, in which young bacteria are given birth to as soon as a fresh nutrient medium is provided. But no one has as yet corroborated Huppe's interesting observations ; on the contrary, laboratory experience with the cholera bacillus has shown it to be one of the most sensitive and non-resistant organism with which we are acquainted. A temperature of 50° is sure death to the germ in a very short time. Its resistance to chemical agents is almost *nil* ; this is especially true with reference to acids ; hence, their destruction by the gastric juice if of normal reaction. Only in an alkaline or neutral culture medium will the cholera germ develop. With reference to dryness, it is also extremely sensitive ; deprived of moisture, they die very quickly. In presence of moisture, on the other hand, they live for months, and in water, sterilized, they multiply luxuriantly. In decomposed fluids the bacilli rapidly lose their existence. Kitasato and Uffelman found this also to be true with reference to artificial intestinal excretions, while Gruber succeeded in obtaining pure cultures from cholera dejections after weeks.

The temperature limits for the germs' development are 42° and 15° C. respectively.

According to the Pettenkofer school, the all-important factor in the occurrence and spread of cholera is the condition of the soil as influenced by season and location.

Heat and moisture, a certain physical consistency and fertility, are the cardinal requirements. The cholera germ, as known, gains entrance to the alimentary tract through the drinking water or food. Any impairment of the digestive function, especially reduction of the acidity of the gastric juice, or a sluggish state of peristalsis, predispose to the disease.

As products of the bacillus there has been isolated a toxalbumine and a toxicpeptone. In addition, cholera cultures form nitritis and indol, which, upon the addition of nitric acid, evolves a red pigment, "cholera red." The latter has been isolated and extracted. Artificial immunity is being tried by Pasteur. A certain natural immunity is conferred by one attack of the disease, but this immunity does not seem to exceed four or five years. Very exceptionally an individual will experience two attacks of cholera during the same epidemic.

